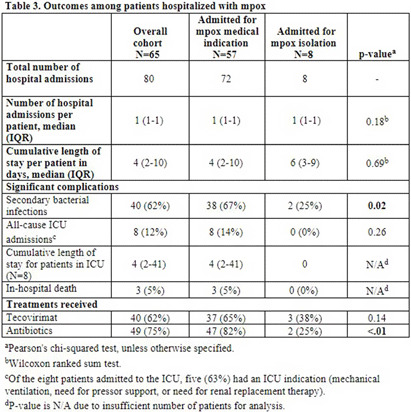# Understanding the impact of mpox-related hospitalizations for medical versus infection control indications in New York City

**DOI:** 10.1017/ash.2024.237

**Published:** 2024-09-16

**Authors:** Katelin Jackson, Eric Lofgren

**Affiliations:** Washington State University

## Abstract

Understanding the impact of mpox-related hospitalizations for medical versus infection control indications in New York City **Background:** New York City (NYC) accounted for 15-20% of new mpox infections at the peak of the 2022-2023 United States outbreak. Globally, 8% of mpox patients required hospitalization. We investigated the proportion of mpox hospitalizations for medical versus infection control indications at two large healthcare systems in the New York metropolitan area. **Methods:** We included all patients admitted to NYU Langone Health or NYC Health + Hospitals for laboratory-confirmed mpox between May 1, 2022, and April 28, 2023. We analyzed demographic information, reasons for hospitalization, length of stay, number and type of co-infections, healthcare encounters, complications, and treatments received. **Results:** Sixty-five patients were hospitalized for mpox, with 8 (12%) admitted primarily for infection control isolation (Table 1). Median age was 35 years (IQR=31-40), 69% were cisgender men, and 38% were Black. Those hospitalized primarily for isolation were more likely to reside in a homeless shelter (50% vs. 9%, p < 0 .01) and less likely to have a private residence (25% vs. 81%, p < 0 .01) than those hospitalized for medical indications. Those hospitalized for medical indications were more likely to have HIV (63% vs. 25%, p=0.04), secondary bacterial infections (67% vs. 25%, p=0.02), and to receive antibiotics (82% vs. 25%, p < 0 .01) (Tables 2 and 3). There was no significant difference in median cumulative length of stay per patient (p=0.69) between those hospitalized for medical versus isolation purposes. Most admissions for medical indications were for soft tissue superinfection (40%), severe pharyngitis and/or proctitis (28%) and pain management (20%). There was no significant difference in the proportion of tecovirimat receipt (65% vs. 38%, p=0.14) between those hospitalized for medical versus isolation purposes. **Conclusion:** Infection control isolation accounted for a significant proportion (12%) of mpox hospitalizations and was associated with a similar median length of stay per patient as hospitalization for medical indications. Our small cohort limits statistical power for comparison between groups. However, our findings argue for increased community-based isolation capacity. This may reduce unnecessary hospitalizations during future outbreaks, particularly amongst unsheltered individuals or those living in congregate settings.

**Disclosure:** Madeline DiLorenzo: Stocks - Abbvie, Amgen Inc., Becton Dickinson, Biogen Inc., Bristol Myers and Squibb, CVS Health, Davita Inc., Elevance Health, Gilead, Henry Schein, Hologic Inc., Humana Inc., Jazz Pharmaceuticals, Laboratory Corp, Merck and Co., Quest Diagnostics, ResMed Inc., Teladoc Health, Vertex Pharmaceuticals, West Pharmaceuticals

**Figure:**
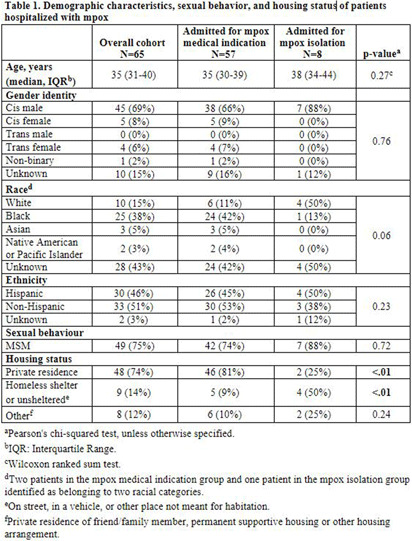

**Figure:**
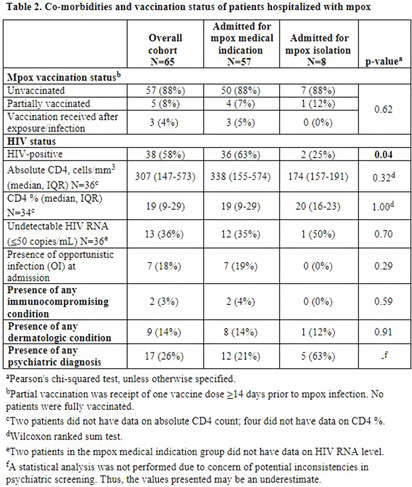

**Figure:**